# Radiosynthesis and First Preclinical Evaluation of the Novel ^11^C-Labeled FAP Inhibitor ^11^C-FAPI: A Comparative Study of ^11^C-FAPIs and (^68^Ga) Ga-DOTA-FAPI-04 in a High–FAP-Expression Mouse Model

**DOI:** 10.3389/fchem.2022.939160

**Published:** 2022-08-05

**Authors:** Cheng Wang, Zhoumi Hu, Fan Ding, Haitao Zhao, Fuqiang Du, Chun Lv, Lianghua Li, Gang Huang, Jianjun Liu

**Affiliations:** ^1^ Department of Nuclear Medicine, Renji Hospital, School of Medicine, Shanghai Jiao Tong University, Shanghai, China; ^2^ Shanghai Key Laboratory of Molecular Imaging, Shanghai University of Medicine and Health Sciences, Shanghai, China

**Keywords:** fibroblast activation protein, fibroblast activation protein inhibitor, carbon-11, 11C-fapis, positron emission tomography

## Abstract

Purpose: ^68^Ga-labeled fibroblast activation protein inhibitors, such as [^68^Ga]Ga-DOTA-FAPI-04 and [^68^Ga]Ga-DOTA-FAPI-46, have been successfully applied in positron emission tomography imaging of various tumor types. To broaden the PET tracers of different positron nuclides for imaging studies of FAP-dependent diseases, we herein report the radiosynthesis and preclinical evaluation of two ^11^C-labeled FAP inhibitors, ^11^C-RJ1101 and ^11^C-RJ1102. Methods: Two phenolic hydroxyl precursors based on a quinoline amide core coupled with a 2-cyanopyrrolidine moiety were coupled with [^11^C]CH_3_I to synthesize ^11^C-RJ1101 and ^11^C-RJ1102. *In vivo* small-animal PET and biological distribution studies of ^11^C-RJ1101 and ^11^C-RJ1102 compared to [^68^Ga]Ga-DOTA-FAPI-04 were conducted in nude mice bearing U87MG tumor xenografts at 30, 60, and 90min, respectively. Results: ^11^C-RJ1101 and ^11^C-RJ1102 were synthesized in over 15% radiochemical yields, with specific activities of 67 GBq/μmol and 34 GBq/μmol, respectively, at the end of synthesis and radiochemical purities greater than 99%. In U87MG tumor xenograft PET studies, the three tracers experienced higher specific uptake at the tumor site. However, because of significant differences in metabolism and clearance, [^68^Ga]Ga-DOTA-FAPI-04 experienced high uptake in the kidney, whereas ^11^C-RJ1101 and ^11^C-RJ1102 showed high uptake in the liver and intestine. Biodistribution studies revealed significant hepatobiliary excretion of ^11^C-RJ1101 and ^11^C-RJ1102. 11C-RJ1102 showed higher specific tumor uptake in U87MG xenografts (1.71 ± 0.08% injected dose per Gram of tissue [ID/g]) than ^11^C-RJ1101 (1.34 ± 0.10%ID/g) and [^68^Ga]Ga-DOTA-FAPI-04 (1.29 ± 0.04%ID/g) after 30 min p. i. In orthotopic glioma models, the uptake values were 0.07 ± 0.03% ([^68^Ga]Ga-DOTA-FAPI-04) and 0.16 ± 0.03% (^11^C-RJ1102), respectively. Conclusion: ^11^C-RJ1101 and ^11^C-RJ1102 are interesting candidates for translation to the clinic, taking advantage of the shorter half-life and physical imaging properties of C-11.

## Introduction

Fibroblast activation protein (FAP) is highly expressed in the stroma of a vast majority of epithelial tumors, as well as in fibrosis and rheumatoid arthritis (Kalluri, 2016). Quinoline amide core-based FAP inhibitors (FAPIs) specifically bind to the enzymatic domain of FAP with nanomolar affinity and high selectivity (Jansen et al., 2013; Jansen et al., 2014; Lindner et al., 2018). More generally, these quinoline amide core-based FAP ligands have been conjugated with bifunctional chelating agents (BCAs), such as 1,4,7,10-tetraazacyclododecane-tetraacetic acid (DOTA) (Loktev et al., 2018; Giesel et al., 2019; Kratochwil et al., 2019) and 1,4,7-triazacyclononane-triacetic acid (NOTA) (Giesel et al., 2021; Wang et al., 2021) V, for radiolabeling with various radiometals. The affinities of the inhibitors to FAP did not change considerably upon coupling of these BCAs. Labeled with the positron emitter radionuclide ^68^Ga, ^18^F-Al, or ^64^Cu complex, these tracers demonstrated high tumor-to-noise contrast ratios, fast elimination, and the successful imaging of tumor metastases (Chen et al., 2020; Watabe et al., 2020; Archibald and Allott, 2021; Giesel et al., 2021; Wang et al., 2021). In addition, the BCA-coupled compounds can be labeled with therapeutic nuclides, such as ^90^Y (Ferdinandus et al., 2021; Rathke et al., 2021), ^153^Sm (Kratochwil et al., 2021), ^177^Lu (Ballal et al., 2021; Kuyumcu et al., 2021; Fu et al., 2022), ^211^At (Ma et al., 2022), and ^225^Ac (Liu et al., 2022), for tumor treatment. Therefore, these compounds have excellent application prospects in clinical nuclear medicine for the diagnosis and treatment of most varieties of tumors. In addition, instead of using BCAs, the glucose analog can be directly labeled with ^18^F and then coupled with a quinoline ring derivative by click chemistry to generate a molecular probe of the ^18^F-labeled FAPI derivative. Preclinical biological evaluation was carried out. However, the uptake of radioactivity in the bone joints indicates that the imaging agent became defluorinated *in vivo* (Toms et al., 2020). However, for the radionuclide ^11^C, one of the commonly used radionuclide in the clinic besides ^18^F, no ^11^C-labeled compounds targeting FAP have been reported. Thanks to its short half-life (20 min), patients can scan two kinds of positron molecular probes in 1 day. This greatly reduces the patient’s waiting time for PET examination.

Herein, we report two ^11^C-labeled small-molecule inhibitors of FAP prepared from precursors based on a quinoline amide core coupled to a 2-cyanopyrrolidine moiety: (*S*)-*N*-(2-(2-cyanopyrrolidin-1-yl)-2-oxoethyl)-6-hydroxyquinoline-4-carboxamide and (*S*)-*N*-(2-(2-cyano-4,4-difluoropyrrolidin-1-yl)-2-oxoethyl)-6-hydroxyquinoline-4-carboxamide. Then, these two precursors were conjugated with [^11^C] CH_3_I to synthesize (*S*)-*N*-(2-(2-cyanopyrrolidin-1-yl)-2-oxoethyl)-6-(methoxy-11C)quinoline-4-carboxamide (^11^C-RJ1101) and (*S*)-*N*-(2-(2-cyano-4,4-difluoropyrrolidin-1-yl)-2-oxoethyl)-6-(methoxy-11C) quinoline-4-carboxamide (^11^C-RJ1102). We report biodistribution studies and small-animal PET studies of these new ^11^C-methylated FAPIs compared with [^68^Ga] Ga-DOTA-FAPI-04 in nude mouse xenografts of FAP-positive tumors.

## Materials and Methods

### General

All chemicals and reagents were commercially obtained from Sigma–Aldrich (Shanghai, China), Bidepharm (Shanghai, China), Huayi (Changshu, China), and TanzhenBio (Nanchang, China) and used without further purification unless otherwise stated. All synthesized compounds were characterized by ^1^H NMR spectroscopy using a 400 MHz Bruker Avance II spectrometer. LC/MS was conducted on an Infinity Lab mass spectrometer connected to an Agilent 1260 series instrument with an Extend-C18 column (50 mm × 2.1 mm, 1.8 μm) at a UV wavelength of 254 nm. ^11^C-CO_2_ was synthesized using a medical cyclotron (HM-10, Sumitomo Heavy Industries Ltd., Tokyo, Japan) with high radiochemical purity (≥ 99%). ^11^C-RJ1101 and ^11^C-RJ1102 were automatically synthesized using a multipurpose synthesizer with a PU-2086 Plus intelligent preparation pump, a UV-2075 Plus intelligent UV/VIS detector, and a OKEN S1729 radioactivity detector (CFN-MPS200, Sumitomo Heavy Industries, Ltd., Japan) and a reverse-phase high-performance liquid chromatography (HPLC) column (C18-B, 250 mm × 10 mm, 5 μm, 120 Å, Morhchem Technologies Inc., United States). The probes’ quality controls were analyzed by HPLC (Agilent 1260 series, United States) on a C18 column (C18-B, 250 mm × 4.6 mm, 5 μm, 100 Å, Morhchem Technologies Inc., United States) with a 1260 Quat pump VL, a 1260 DAD VL detector, a 1260 Vialsampler automatic sample injector, and an additional γ-detector (Eckert and Ziegler, United States). The gradient and flow rate of the water/acetonitrile mobile phase were modified for the individual products. The radioactivity was measured using a CRC®-55T activity meter (The China National Nuclear Corporation, Beijing, United States). Micro-PET/CT imaging was performed on an IRIS PET/CT system (Inviscan Imaging Systems, France).

The two precursors [(*S*)-*N*-(2-(2-cyanopyrrolidin-1-yl)-2-oxoethyl)-6-hydroxyquinoline-4-carboxamide and (*S*)-*N*-(2-(2-cyano-4,4-difluoropyrrolidin-1-yl)-2-oxoethyl)-6-hydroxyquinoline-4-carboxamide], as well as two corresponding standard compounds [(*S*)-*N*-(2-(2-cyanopyrrolidin-1-yl)-2-oxoethyl)-6-(methoxy)quinoline-4-carboxamide and (*S*)-*N*-(2-(2-cyano-4,4-difluoropyrrolidin-1-yl)-2-oxoethyl)-6-(methoxy)quinoline-4-carboxamide], were designed and synthesized by TanzhenBio and Huayi. Reagents including phosphate-buffered saline (PBS), cell culture medium, fetal bovine serum (FBS), penicillin–streptomycin solution (PS), GlutaMAX, nonessential amino acids (NEAAs), and trypsin for cell culture and subsequent experiments were obtained from Gibco (Thermo Fisher Scientific, China).

### Radiosynthesis of ^11^C-RJ1101, ^11^C-RJ1102, and [^68^Ga] Ga-DOTA-FAPI-04

The radiosynthesis of ^11^C-RJ1101 and ^11^C-RJ1102 was performed in a multipurpose synthesizer. Briefly, no-carrier-added [^11^C] CO_2_ was produced using an HM-10 cyclotron via the ^14^N (p,α)^11^C reaction and a gaseous target of N_2_ (+ 0.5% O_2_). Using the liquid method, [^11^C] CO_2_ was trapped in cold lithium aluminum hydride [LAH, -15 C in 0.1 M dry tetrahydrofuran (THF)], giving rise to the reduced product [^11^C] CH_3_OH. The THF was then evaporated off under a constant stream of N_2_, and 0.5 ml of 67% HI was added to the residue containing [^11^C] CH_3_OH. The vessel was sealed and heated to 180°C before exposing the solution to a stream of nitrogen gas that bubbled the [^11^C] CH_3_I product into another -10°C sealed vessel containing 250 μl of *N*,*N*-dimethylformamide (DMF), 1 mg of precursor, and 5 μl of 5 M NaOH. The homogeneous pale-yellow solution was heated at 55 C for 2–5 min, and then the reaction mixture was neutralized and diluted by 1.5 ml of HPLC solution and separated by semipreparative HPLC. The final product was trapped in a C18 Sep-Pak cartridge (WAT020515, Waters) and eluted with 0.5 ml of ethanol. After formulation with 4.5 ml of sterile normal saline and subsequent filtration with 0.22-µm filter membranes, the ^11^C-labeled molecular probes were used for the next experiments. The probes’ quality controls were analyzed by HPLC, for ^11^C-RJ1101. The chromatographic conditions were described below: flow rate of 1 ml/min, UV of 254 nm, injection volume of 20 μl, mobile phase A (0.1 M ammonium formate aqueous solution + 0.5% acetic acid), and mobile phase B (acetonitrile), 30%B in 15 min. And for ^11^C-RJ1102, the mobile phase was 37% B in 15 min. The radiosynthesis of [^68^Ga] Ga-DOTA-FAPI-04 is illustrated in the literature (Lindner et al., 2018; Loktev et al., 2018). Briefly, 100 μg of DOTA-FAPI-04 precursor and 4 ml of a [^68^Ga]GaCl_3_ eluent solution (0.37 GBq in 0.05 M HCl) were mixed and adjusted to pH 4.0–4.5 with 1 ml of sodium acetate (0.25 M in water). After heating to 100°C for 10 min under constant shaking (600 rpm), the product was isolated by a C18 Sep-Pak cartridge using ethanol (0.5 ml) as the eluent. After formulation with 4.5 ml sterile normal saline and filtration through 0.22-µm filter membranes, [^68^Ga] Ga-DOTA-FAPI-04 was used for the subsequent experiments, and the radiochemical purity was assessed by HPLC (Supplementary Figure S14).

The ^11^C-labeled compounds ^11^C-RJ1101 and ^11^C-RJ1102 were incubated in PBS at 37°C for 30, 60, 90, 120, and 150 min to measure the *in vitro* stability. The radiochemical purities were analyzed by a radio-HPLC column (C18-B, 250 mm × 4.6 mm, 5 μm, 120 Å, Morhchem Technologies Inc., United States) (details presented in the Supplementary Material).

### Tumor-Bearing Mouse Model Establishment

All animal experimental procedures and protocols were approved by the Institutional Animal Care and Use Committee (Renji Hospital, School of Medicine, Shanghai Jiao Tong University). For subcutaneous models, U87MG cells (2 × 10^6^ cells in 100 μl PBS) were implanted into the right flank of female BALB/c nu/nu athymic mice to establish subcutaneous tumor xenografts. For orthotopic xenografts, U87MG cells (0.5 × 10^6^ cells in 100 μl PBS) were transplanted into the brains of female BALB/c nude mice (4–6 weeks old). The subcutaneous and orthotopic models were ready for PET imaging 3 weeks after tumor cell inoculation.

### PET Imaging and Data Analyses

The products of ^11^C-RJ1101, ^11^C-RJ1102, and [^68^Ga] Ga-DOTA-FAPI-04 were diluted or concentrated to a 74 MBq/mL. Then, 7.4 MBq (approximately 0.1 ml) of ^11^C-RJ1101, ^11^C-RJ1102, or [^68^Ga] Ga-DOTA-FAPI-04 was intravenously injected into U87MG tumor-bearing nude mice (*n* = 3 for each group). For static PET imaging, the acquisition times were 30, 60, and 90 min postinjection (p.i.). Each static scan consists of CT scan and PET scan. The CT scan time is 20 s and the PET scan time is 10 min. For dynamic PET imaging, the duration of the scan was 60 min, and the reconstruction frames were 12 × 10 s, 6 × 30 s, and 11 × 300 s. Regions of interest (ROIs) in the tumor, liver, heart, kidney, brain, and lung were counted on the PET images to quantify the radioactive signals. For orthotopic xenografts, scans of ^11^C-RJ1102 and [^68^Ga] Ga-DOTA-FAPI-04 were performed at 60 min p. i. All PET/CT imaging experiments were performed on an IRIS small animal PET/CT imaging system (inviscan SAS, Strasbourg, France). PET data were reconstructed with three-dimensional ordered-subset expectation-maximization (3D-OSEM) algorithm with Monte-Carlo based accurate detector model and subsequently was analyzed with Avatar 1.2 software (Pingseng China).

### Biodistribution Studies

In the biodistribution study, the products ^11^C-RJ1101 and ^11^C-RJ1102 were also diluted to a concentration of 74 MBq/ml. U87MG mice were injected with 3.7 MBq of each tracer and sacrificed at different times (30, 60, and 90 min p.i.; *n* = 3 for each time point). The main organs, including the kidney, intestine, liver, heart, lung, muscle, brain, and tumors, were isolated, weighed, and analyzed. The biodistribution in the [^68^Ga] Ga-DOTA-FAPI-04 group (3.7 MBq) with approximately 100 μg of unlabeled DOTA-FAPI-04 was also evaluated for comparison. The radioactivity of the samples was determined using an automated γ-counter (Perkin-Elmer), and the uptake of the radiotracers in different organs/tissues was calculated and presented as percent of injected dose per Gram of tissue (%ID/g, mean ± SD).

### Statistical Analysis

Statistical analysis was performed using GraphPad software. Data are presented as the means ± SD as stated in the figure legends. *p* < 0.05 was considered statistically significant.

## Results

### Synthesis and Radiolabeling

**FIGURE 1:**
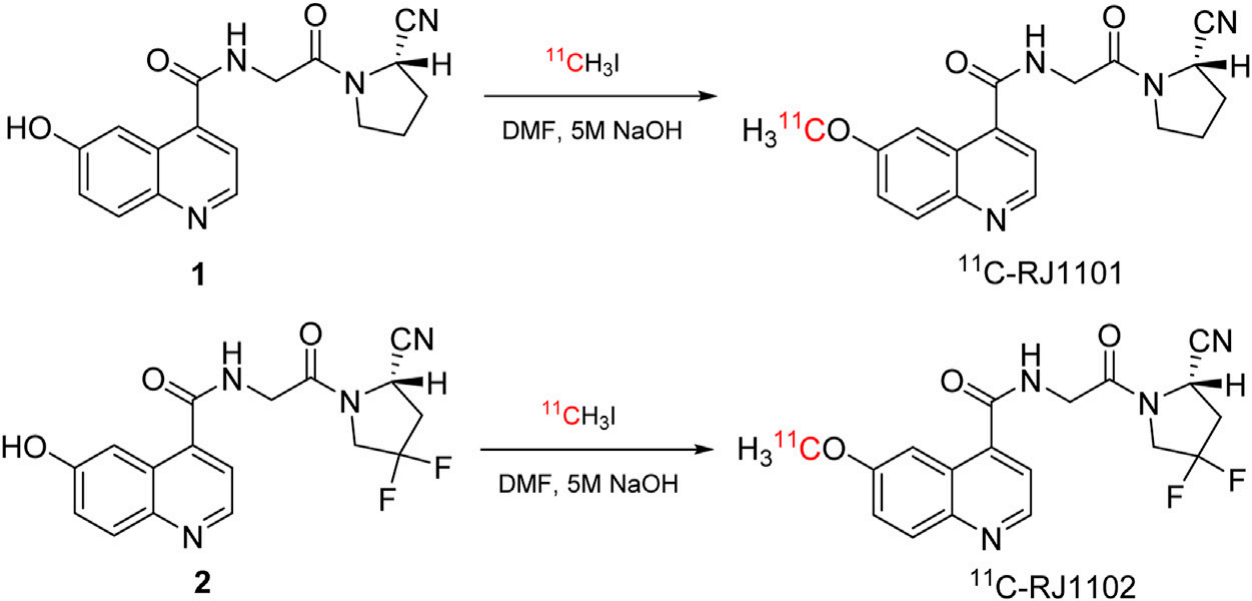
The radiolabeling of ^11^C-RJ1101 and ^11^C-RJ1102.

Two precursor compounds, **1** and **2**, and two standards compounds, **3** and **4**, were synthesized by conjugation of amino and carboxyl groups (Supplementary Figure S1). The LC/MS and ^1^H NMR spectroscopic data confirmed formation of the desired compounds (Supplementary Figures S2–S9). The radiolabeling of ^11^C-RJ1101 and ^11^C-RJ1102 is shown in Figure 1. Using the wet method, [^11^C] CO_2_ (25 ± 3 GBq, *n* = 10) produced by a cyclotron was trapped in LAH (0.1 M in THF). With the addition of hydroiodic acid (w = 67%) and an increase in the reaction temperature, ^11^CH_3_I was generated (14 ± 2 GBq, with radiochemical yields of 56%, *n* = 10) and then bubbled into the yellow solution of the nonradiolabeled precursor in DMF which contained 5 μl of aqueous NaOH solution (*c* = 5 M). After HPLC separation and C18 column concentration, the final tracers ^11^C-RJ1101 (3.2 ± 0.4 GBq, *n* = 5) and ^11^C-RJ1102 (2.2 ± 0.3 GBq, *n* = 5) were generated in radiochemical yields of 23 and 16% (based on ^11^CH_3_I and without decay correction). The specific activities were 67 ± 12 GBq/μmol (*n* = 5) and 34 ± 8 GBq/μmol (*n* = 5), respectively, at the end of synthesis. The total synthesis time was 30 min from the end of bombardment. The radioactive purities of these two tracers were also over 99% in PBS at 150 min, showing that the stabilities were high *in vitro*. The data, including the semipreparative HPLC separation conditions and stability, are shown in the supplemental material (Supplementary Figures S10–S14).

### Small-Animal PET Studies

**FIGURE 2:**
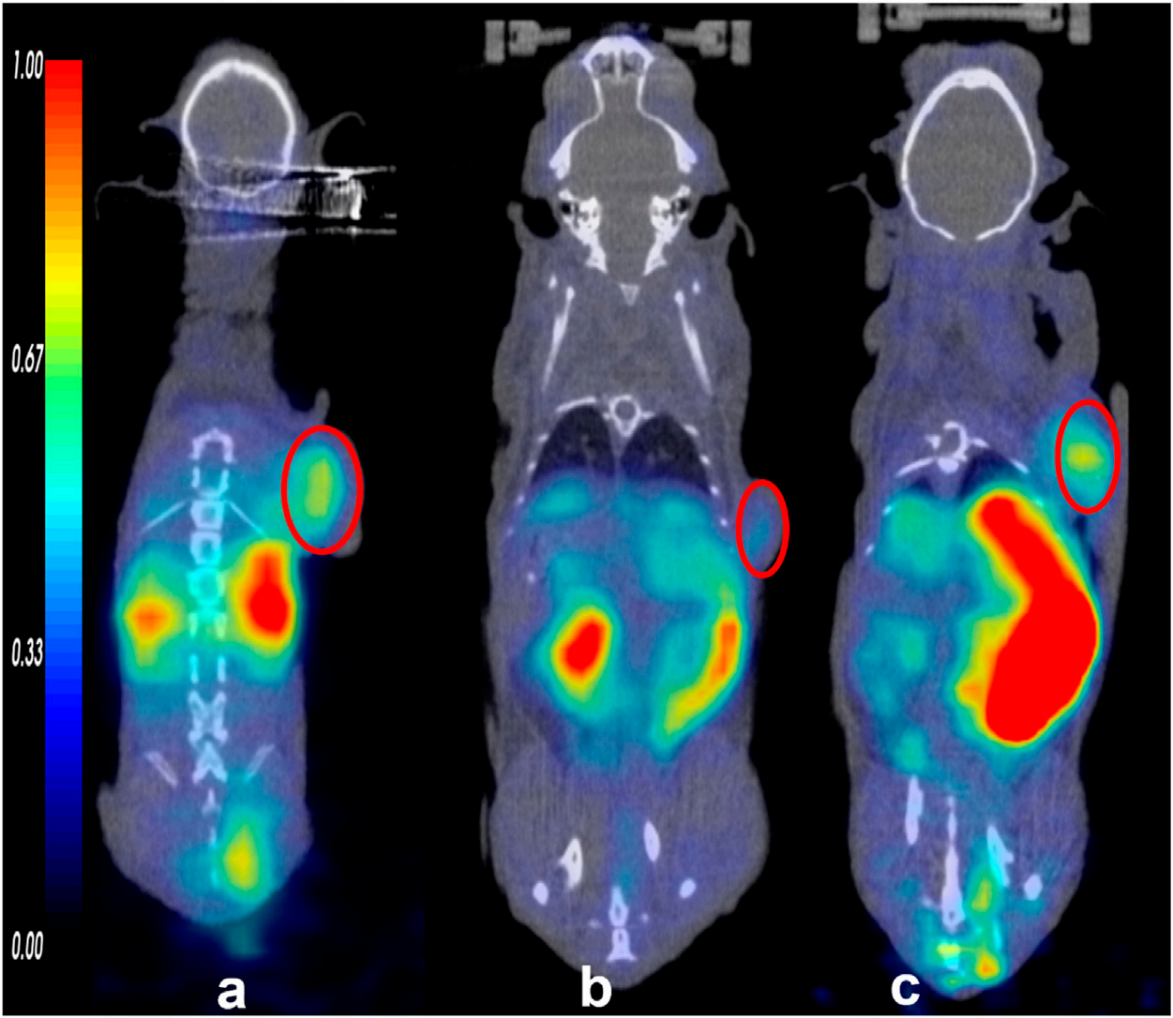
The small animal PET/CT imaging of the tracers (^68^Ga) Ga-DOTA-FAPI-04 **(A)**, ^11^C-RJ1101 **(B)**, and ^11^C-RJ1102 **(C)** at 60 min. And the tumor was indicated by the red circle.

**FIGURE 3:**
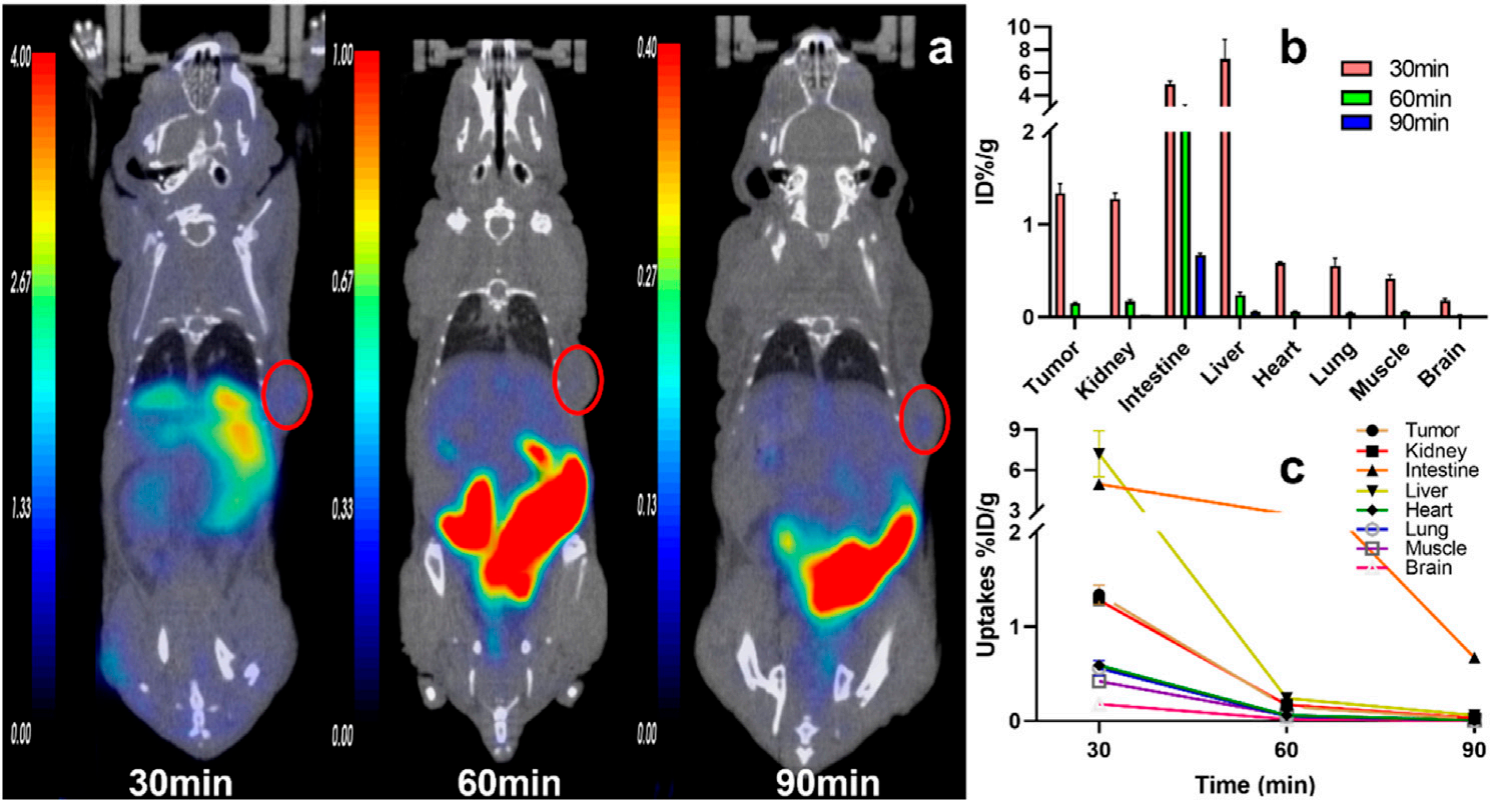
The representative static PET imaging and distribution of ^11^C-RJ1101, **(A)** the representative static PET imaging of ^11^C-RJ1101 at 30, 60, 90 min in U87MG tumor-bearing nude mice. And the tumor was indicated by the red circle; **(B)** the organs or tissues uptakes of ^11^C-RJ1101 in U87MG tumor-bearing nude mice at 30, 60, 90 min; **(C)** the time–active curve (TAC) of ^11^C-RJ1101 at 30, 60, 90 min.

**FIGURE 4:**
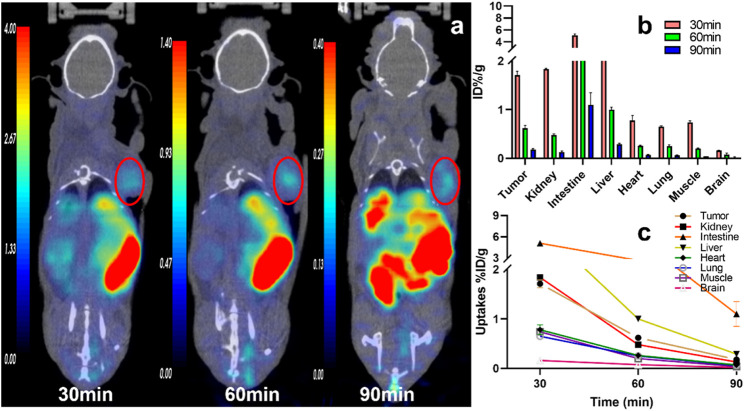
The representative static PET imaging and distribution of ^11^C-RJ1102, **(A)** the representative static PET imaging of ^11^C-RJ1102 at 30, 60, 90 min in U87MG tumor-bearing nude mice. And the tumor was indicated by the red circle; **(B)** the organs or tissues uptakes of ^11^C-RJ1102 in U87MG tumor-bearing nude mice at 30, 60, and 90 min; **(C)** the time–active curve (TAC) of ^11^C-RJ1102 at 30, 60, 90 min.

**FIGURE 5:**
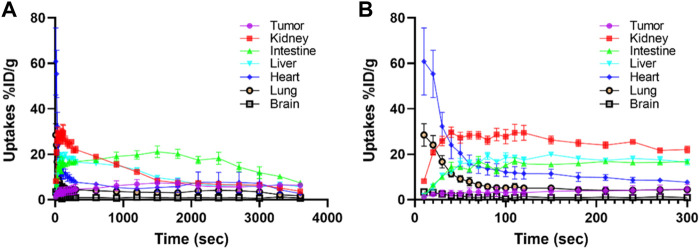
Dynamic time–activity curves of ^11^C-RJ1102 in the heart, kidney, liver, brain, lung, Intestine, and tumor in U87MG tumor-bearing nude mice. The difference between **(A,B)** is the ordinate, for **(A)** the time is from 0 to 3,600 s. And for **(B)** the time is from 0 to 300 s.

**FIGURE 6:**
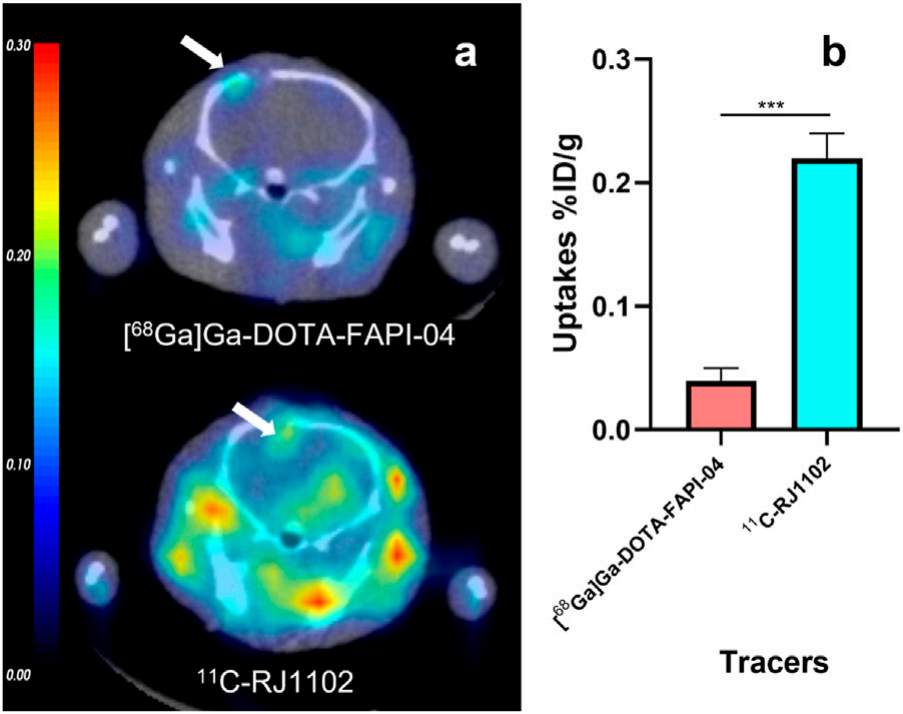
The orthotopic xenograft gliomas model small animal PET of ^11^C-RJ1102 and (^68^Ga) Ga-DOTA-FAPI-04 at 60 min p.i. **(A)** The representative static PET imaging of ^11^C-RJ1102 and (^68^Ga) Ga-DOTA-FAPI-04 at 60 min p.i in U87MG orthotopic xenograft gliomas models. **(B)** The uptakes of ^11^C-RJ1102 and (^68^Ga) Ga-DOTA-FAPI-04 in tumor.

Tissue accumulations of the tracers were described with an ID%/g scale. In U87MG tumor-bearing nude mice, all three tracers were highly absorbed in the tumor at 30 min p. i., and the uptake decreased relatively slowly until 90 min. The accumulation of [^68^Ga]Ga-DOTA-FAPI-04 at different times is shown in the Supplementary Figures S15, S16). The tumor accumulation of ^11^C-RJ1101 and ^11^C-RJ1102 showed results similar to those of [^68^Ga] Ga-DOTA-FAPI-04 at 30 min p. i. However, the greatest differences were observed in the liver and kidney because of the significant difference in lipid-water partition coefficients (log P) at 60 min p. i. (Figure 2). The coronal section images at different scan times for the three tracers are shown in Figure 3 and Figure 4. Like the [^68^Ga] Ga-DOTA-FAPI-04 in U87MG tumor model mice, washout kinetics of ^11^C-RJ1101 are fast in the kidney, the liver, and intestine. However, the clearance of ^11^C-RJ1102 from the liver and intestine was relatively slow. For ^11^C-RJ1102, 60-min dynamic PET was performed in U87MG tumor xenografts. The tumor accumulation of ^11^C-RJ1102 was rapid. In contrast, rapid clearance from the heart, kidney, and liver was observed. Moreover, uptake in the intestine was also high (Figure 5). Regarding the tumor, accumulation of ^11^C-RJ1102 in U87MG tumor xenografts was rapid. Slightly increased tumor uptake was observed from 30 to 60 min, and then constant uptake occurred between 60 and 90 min. In the U87MG orthotopic xenograft glioma model small-animal PET study, ^11^C-RJ1102 accumulated in the tumor at 60 min p. i. significantly higher levels than [^68^Ga] Ga-DOTA-FAPI-04 (0.22 ± 0.02 vs. 0.04 ± 0.01, *n* = 3, *p* = 0.000153, Figure 6).

### Organ Distribution in U87MG Tumor Xenografts

The biodistribution of the three tracers in U87MG tumor xenografts was determined by *ex vivo* counting in tissues collected 30, 60, and 90 min after injection (Table 1). At 30, 60, 120 min p. i., [^68^Ga]Ga-DOTA-FAPI-04 accumulated mainly in the tumor (1.29 ± 0.04, 0.60 ± 0.09 and 0.19 ± 0.05% ID/g) and kidney (28.37 ± 4.67, 1.54 ± 0.38 and 0.46 ± 0.06% ID/g), and the tumor-to-muscle (T/M) ratio was 1.16 ± 0.25, 2.53 ± 0.38 and 4.74 ± 0.07, respectively. Other organs demonstrated low nonspecific binding that quickly decreased, resulting in a low background signal and favorable tumor-to-background ratios.

**TABLE 1:** The distribution of three molecular probes in different organs.

Tracers	(68Ga) Ga-DOTA-FAPI-04			^11^C-RJ1101			^11^C-RJ1102		
**Time(min)**	30	60	120	30	60	90	30	60	90
Tumor	1.29 ± 0.04	0.60 ± 0.09	0.19 ± 0.05	1.34 ± 0.10	0.15 ± 0.01	0.02 ± 0.00	1.71 ± 0.08	0.62 ± 0.06	0.18 ± 0.02
**Kidney**	28.37 ± 4.67	1.54 ± 0.38	0.46 ± 0.06	1.28 ± 0.06	0.17 ± 0.02	0.03 ± 0.00	1.84 ± 0.01	0.48 ± 0.02	0.13 ± 0.02
**Intestine**	1.40 ± 0.03	0.67 ± 0.05	0.27 ± 0.02	4.95 ± 0.31	2.72 ± 0.45	0.67 ± 0.02	5.08 ± 0.24	2.63 ± 0.28	1.10 ± 0.25
**Liver**	2.08 ± 0.05	0.90 ± 0.07	0.22 ± 0.03	7.20 ± 1.71	0.24 ± 0.03	0.06 ± 0.00	2.87 ± 0.12	1.00 ± 0.05	0.29 ± 0.02
**Heart**	2.86 ± 0.09	0.43 ± 0.03	0.08 ± 0.01	0.59 ± 0.01	0.06 ± 0.01	0.01 ± 0.00	0.78 ± 0.10	0.26 ± 0.01	0.07 ± 0.01
**Lung**	1.69 ± 0.07	0.27 ± 0.01	0.10 ± 0.01	0.56 ± 0.08	0.05 ± 0.01	0.01 ± 0.00	0.65 ± 0.02	0.25 ± 0.03	0.06 ± 0.01
**Muscle**	1.15 ± 0.28	0.23 ± 0.01	0.04 ± 0.01	0.42 ± 0.04	0.06 ± 0.01	0.01 ± 0.00	0.74 ± 0.03	0.20 ± 0.01	0.04 ± 0.00
**Brain**	0.52 ± 0.03	0.09 ± 0.02	0.02 ± 0.01	0.18 ± 0.02	0.02 ± 0.00	0.00 ± 0.00	0.16 ± 0.01	0.07 ± 0.03	0.02 ± 0.01

**FIGURE 7:**
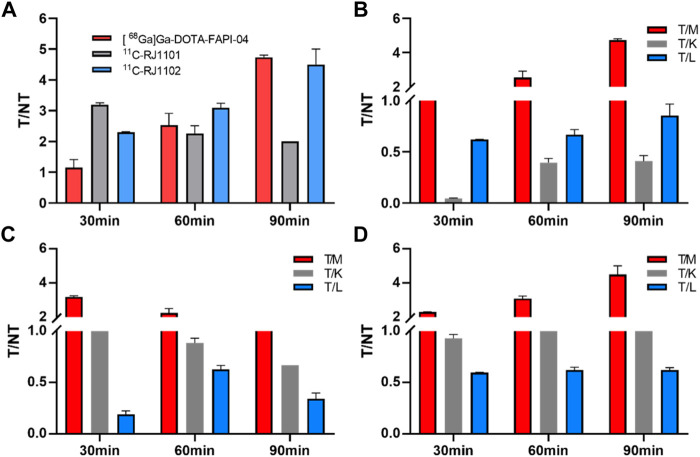
The T/NT ratios in U87MG tumor-bearing nude mice of (^68^Ga) Ga-DOTA-FAPI-04, ^11^C-RJ1101, and ^11^C-RJ1102. **(A)** the T/M ratios of (^68^Ga) Ga-DOTA-FAPI-04, ^11^C-RJ1101 and ^11^C-RJ1102 after 30, 60, and 90 min p.i. **(B)** the T/M, T/K, and T/L ratios of (^68^Ga) Ga-DOTA-FAPI-04 after 30, 60, and 90 min p.i. **(C)** the T/M, T/K, and T/L ratios of ^11^C-RJ1101 after 30, 60, and 90 min p.i. **(D)** the T/M, T/K, and T/L ratios of ^11^C-RJ1102 after 30, 60, and 90 min p.i.

At 30, 60, 90 min p. i., ^11^C-RJ1101 accumulated mainly in the tumor (1.34 ± 0.10, 0.15 ± 0.01 and 0.02 ± 0.00% ID/g), kidney (1.28 ± 0.06, 0.17 ± 0.02 and 0.03 ± 0.00% ID/g), and liver (7.20 ± 1.27, 0.24 ± 0.03 and 0.06 ± 0.00% ID/g), and the T/M ratio was 3.19 ± 0.06, 2.26 ± 0.25, and 2.00 ± 0.00, respectively, (Figure 7). The higher uptake in the liver and intestine demonstrated a slow decrease and clearance, resulting in a higher background signal but a more favorable T/M ratio than [^68^Ga] Ga-DOTA-FAPI-04 (Figure 7).

At 30, 60, 90 min p. i., ^11^C-RJ1102 accumulated mainly in the tumor (1.71 ± 0.08, 0.62 ± 0.06 and 0.18 ± 0.02%ID/g), kidney (1.84 ± 0.01, 0.48 ± 0.02 and 0.13 ± 0.02%ID/g), and liver (2.87 ± 0.12, 1.00 ± 0.05 and 0.29 ± 0.02%ID/g). The T/M ratios were 2.30 ± 0.32, 3.10 ± 0.14, and 4.50 ± 0.50, respectively (Figure 7). The higher uptake of ^11^C-RJ1102 in the liver and intestine was similar to that of ^11^C-RJ1101. Additionally, slowly decreasing clearance resulted in a higher background signal but also a more favorable T/M ratio than those of [^68^Ga] Ga-DOTA-FAPI-04.

## Discussion

Based on quinoline amide core-based FAP inhibitors, many macrocyclic species, such as DOTA-, NOTA-conjugated chelation reagents, have been developed (Windisch et al., 2021). However, these bifunctional chelator-coupled compounds have been labeled with metal radionuclides, such as ^68^Ga and Al^18^F for PET imaging, ^99m^Tc for SPECT imaging, and ^177^Lu, ^225^Ac, and ^223^Ra for internal exposure treatment in the clinic. Among the imaging tracers, [^68^Ga]Ga-DOTA-FAPI-04 showed excellent pharmacokinetic mechanisms for clinical use in a variety of tumors. As derivatives of small-molecule inhibitors, these tracers showed quick metabolism and clearance from the body, resulting in excellent T/NT values and lower total-body effective doses. Due to the rapid biological half-lives, the short physical half-life of ^68^Ga (67.7 min), and the high specificity, [^68^Ga]Ga-DOTA-FAPI-04 is the most studied and reported PET molecular imaging probe targeting FAP recently.

In this research, we chose the exact inhibitor [(S)-*N*-(2-(2-cyano-4,4-difluoropyrrolidin-1-yl)-2-oxoethyl)-6-(methoxy) quinoline-4-carboxamide, IC_50_ = 8.5 ± 0.9 nM (3)] of FAP, and its analogue (S)-*N*-(2-(2-cyanopyrrolidin-1-yl)-2-oxoethyl)-6-(methoxy) quinoline-4-carboxamide as the standard compounds, labeled them with the PET radionuclide ^11^C (half-life, 20.4 min), and examined the potential of the tracers as PET molecular imaging probes in tumor model mice.

First, the precursors of these inhibitors were synthesized and labeled with ^11^CH_3_I or ^11^CH_3_OTf in several organic solvents, such as DMF, DMSO, THF, and acetone, containing different bases, such as NaOH, NaH, and triethylamine. The reaction temperature was controlled at room temperature, 55 C or 80 C. Finally, the optimal reaction conditions were 1 mg of precursor, ^11^CH_3_I in 250 µl of DMF containing 5 µl of NaOH (5 M) at 55°C, resulting in radiochemical yields of 23% (^11^C-RJ1101) and 16% (^11^C-RJ1102). The reaction mixture was purified by semi-HPLC and concentrated by solid-phase extraction (SPE). Immediately after, the final product was eluted by anhydrous ethanol, diluted with sterile normal saline, and identified by HPLC with a radio detector.

The significant differences in log P (which was simulated by ADMETlab 2.0 database) among the three radioprobes reflected the different metabolism and clearance pathways. The higher water solubility of [^68^Ga]Ga-DOTA-FAPI-04 (log *p* = 0.25) resulted in high kidney clearance, and the higher lipid solubility of ^11^C-RJ1101 (log *p* = 0.63) and ^11^C-RJ1102 (log *p* = 1.27) resulted in good hepatobiliary clearance. These results were confirmed by small-animal PET/CT imaging and the organ distributions in the model mice.

As previously described, [^68^Ga] Ga-DOTA-FAPI-04 was rapidly distributed in all organs of the model mice in 30 min. Because of the rapid distribution and metabolism clearance from kidney, high specificity to the FAP, and the high stability of the probe *in vivo*, the T/K ratios showed a consistent increase from 0.04 ± 0.01 (30 min p.i.) to 0.41 ± 0.06 ID%/g (after 90 min p. i.), and the corresponding tumor/muscle (T/M) ratios changed from 1.16 ± 0.25 to 4.74 ± 0.07.

Similar to the [^68^Ga] Ga-DOTA-FAPI-04 small-animal imaging and organ distribution in U87MG cells, both ^11^C-RJ1101 and ^11^C-RJ1102 were rapidly distributed in all organs of the model mice in 30 min. The T/K ratios for these two probes were 1.05 ± 0.03 and 0.93 ± 0.04 at 60 and 90 min p.i., and the T/K radios were 0.89 ± 0.05 and 0.67 ± 0.01 and 1.29 ± 0.07 and 1.39 ± 0.06, respectively. Especially, ^11^C-RJ1102 showed higher specific tumor uptake than [^68^Ga] Ga-DOTA-FAPI-04 at 30 min p.i. (1.71 ± 0.08%, *n* = 3 vs. 1.29 ± 0.04, *n* = 3; *p* = 0.0012). However, the greatest difference was that the organ with the highest uptake was the kidney for the previous tracer but the liver for ^11^C-RJ1101 and ^11^C-RJ1102. Both ^11^C-RJ1101 and ^11^C-RJ1102 accumulated rapidly in the tumor and other organs in 30 min and resulted in higher T/M (3.19 ± 0.06 in ^11^C-RJ1101 and 2.30 ± 0.02 in ^11^C-RJ1102). As the liver is the major organ that metabolizes most drugs, the T/L ratios were examined. For ^11^C-RJ1101, the ratios at 30, 60, and 90 min p.i. were 0.19 ± 0.03, 0.63 ± 0.04, and 0.34 ± 0.06, respectively. For ^11^C-RJ1102, the ratios were 0.60 ± 0.03, 0.62 ± 0.03, and 0.62 ± 0.03. Significant disparities in the liver and kidney were observed because of the differences in drug metabolism and clearance. As a difference between [^68^Ga]Ga-DOTA-FAPI-04 and ^68^Ga-RJ1102, due to the existence of difluorine atoms in the proline derivative residues of ^11^C-RJ1102, the lipid solubility was enhanced compared with that of ^11^C-RJ1101, resulting in rapid accumulation and slow clearance in organs, especially in tumors.

The *in vivo* distribution experiment of the above tumor model showed that ^11^C-RJ1102 had stronger lipid solubility. Therefore, the brain uptake of ^11^C-RJ1102 and [^68^Ga]Ga-DOTA-FAPI-04 was evaluated in the mouse model of glioma *in situ* in the follow-up experiment. An interesting finding was that in contrast to the radioactive accumulations in the brain of U87MG tumor xenografts and in the orthotopic model, no differences were observed between the two at 60 min p. i. except for with ^11^C-RJ1102. A significant difference in the brain was observed at 60 min p. i. In addition to differences in uptake in glioma *in situ*, there were also significant differences in the uptake of the two molecular probes in other parts of the brain. The likely reason is that the blood–brain barrier is disrupted, resulting in significant uptake of molecular probes in the brain. At the same time, due to the great difference in the lipid solubility of [^68^Ga]Ga-DOTA-FAPI-04 and ^11^C-RJ1102, the metabolism of ^11^C-RJ1102 in other regions of the brain is different. Further studies of this observed phenomenon are ongoing.

## Conclusion

Although different from tissue absorption and clearance with [^68^Ga]Ga-DOTA-FAPI-04, the ^11^C-labeled FAP inhibitors ^11^C-RJ1101 and ^11^C-RJ1102 experience similar tumor uptake and longer tumor retention times. These two ^11^C-labeled FAPIs are interesting candidates for translation to the clinic, taking advantage of the shorter half-life and physical imaging properties of C-11.

## Data Availability

The datasets presented in this study can be found in online repositories. The names of the repository/repositories and accession number(s) can be found in the article/Supplementary Material.
