# Supplementary material for: Radiosynthesis and First Preclinical Evaluation of the Novel 11C-Labeled FAP Inhibitor 11C-FAPI: A Comparative Study of 11C-FAPIs and (68Ga) Ga-DOTA-FAPI-04 in a High–FAP-Expression Mouse Model
Source: Front Chem. 2022 Aug 5;10:939160. doi: 10.3389/fchem.2022.939160 (PMC9388731; doi:10.3389/fchem.2022.939160)
Supplement: Supplementary file 1 [file DataSheet1.docx]

## Supplemental materials

### Procedures of the two precursors compound 1, compound 2, and two standards compound 3, compound 4

The general coupling reactions were compound 5 (6-hydroxyquinoline-4-carboxylic acid, 1 eq.) or compound 8 (6-methoxyquinoline-4-carboxylic acid, 1 eq.) react with N, N-diisopropylethylamine (DIPEA, 1.2 eq.), N-hydroxy-benzotriazole (HOBt, 1.2 eq.), and 2-(1Hbenzotriazole-1-yl)21,1,3,3-tetramethyluronium hexafluorophosphate (HBTU, 1.9 eq.) in an ice-water bath for two minutes, and then compound 6 ((S)-1-glycylpyrrolidine-2-carbonitrile, 1.3 eq.) or compound 7 ((S)-4,4-difluoro-1-glycylpyrrolidine-2-carbonitrile, 1.3 eq.) were added in the above solution, respectively. The reaction was carried out at room temperature for 2 h. Then the four products were collected by chromatographic separations which were performed on a silica gel column. The LC/MS and ^1^H NMR data were illustrated in Supplemental Figure 2-9.

### Supplemental Figures

Figure S1. Procedures of the precursors and standards.


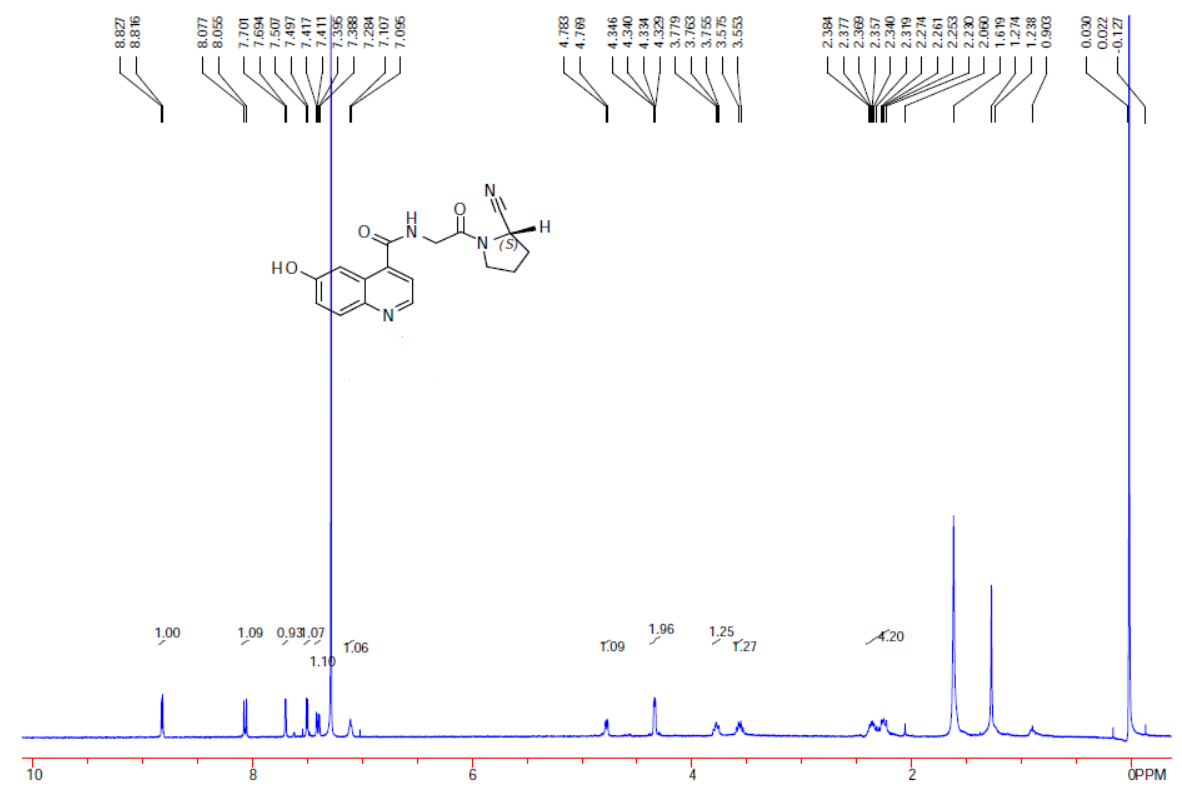


Figure S2. ^1^H NMR spectroscopic data for compound **1**.


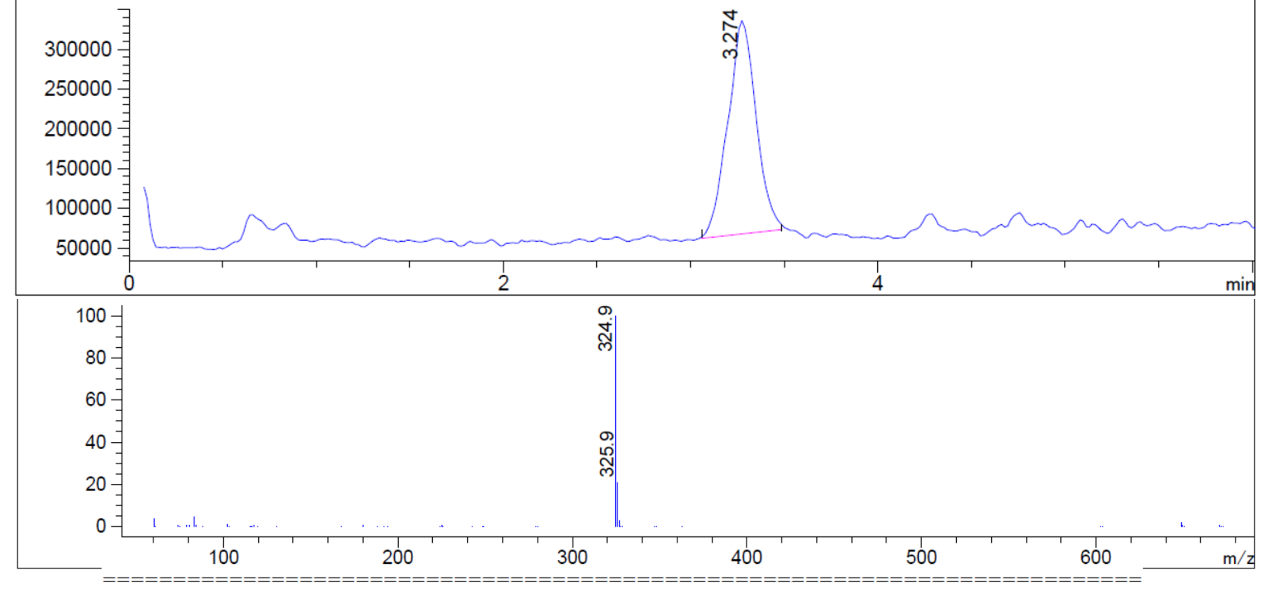


Figure S3. LC/MS spectroscopic data for compound **1**.


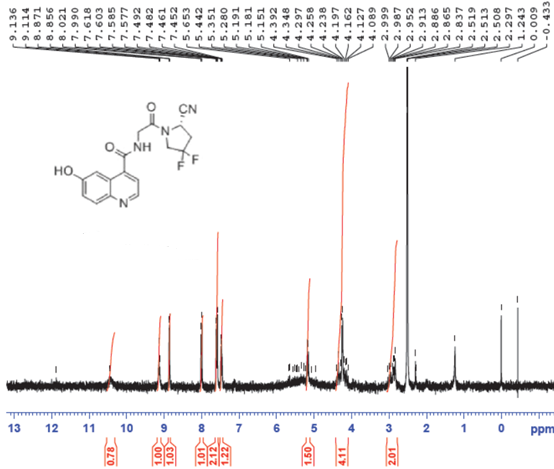


Figure S4. ^1^H NMR spectroscopic data for compound **2**.


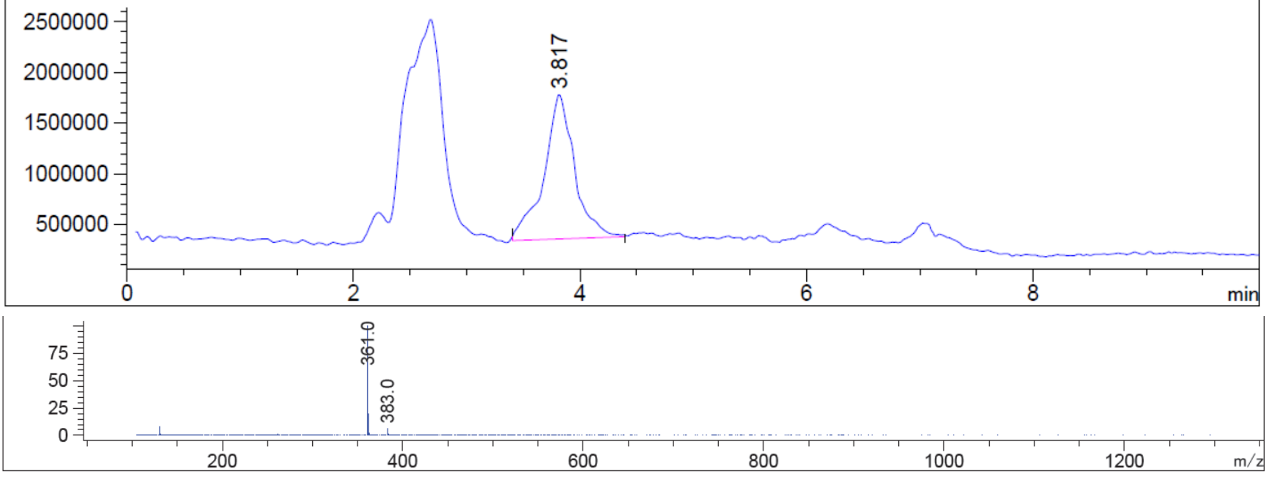


Figure S5. LC/MS spectrometry for compound **2**.

**
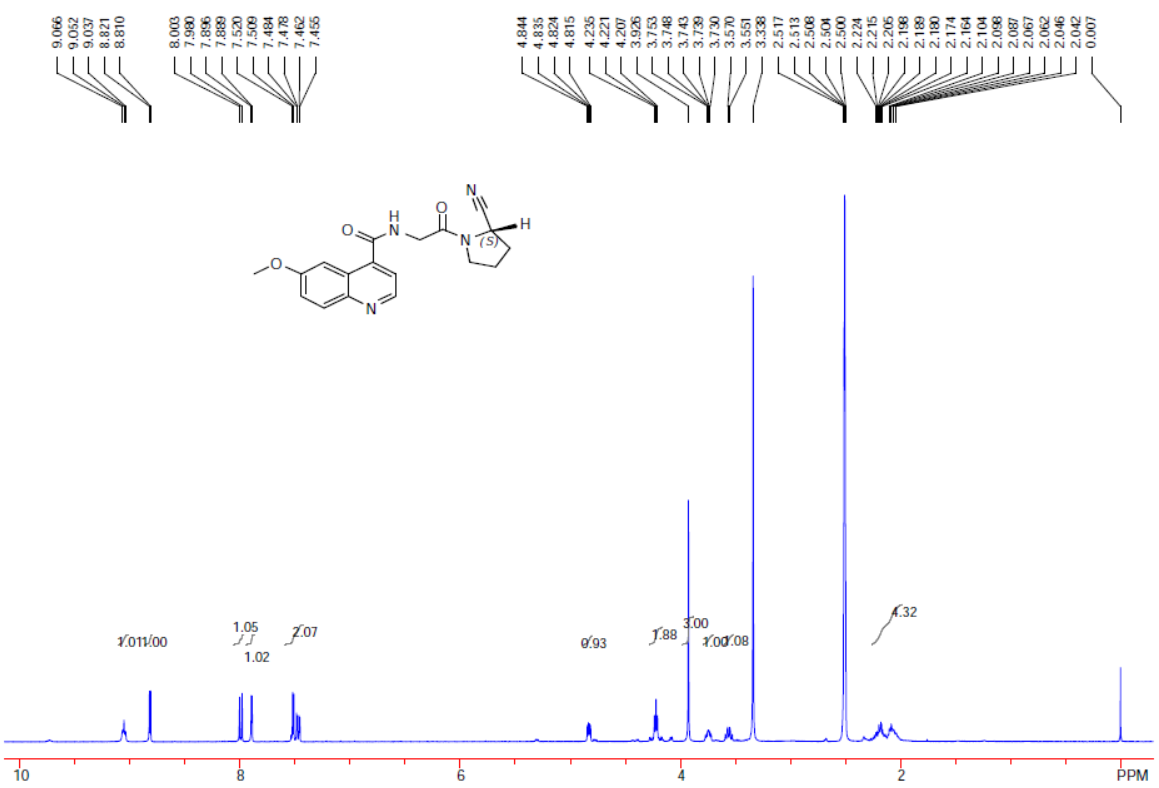
**

Figure S6. ^1^H NMR spectroscopic data for compound **3**.


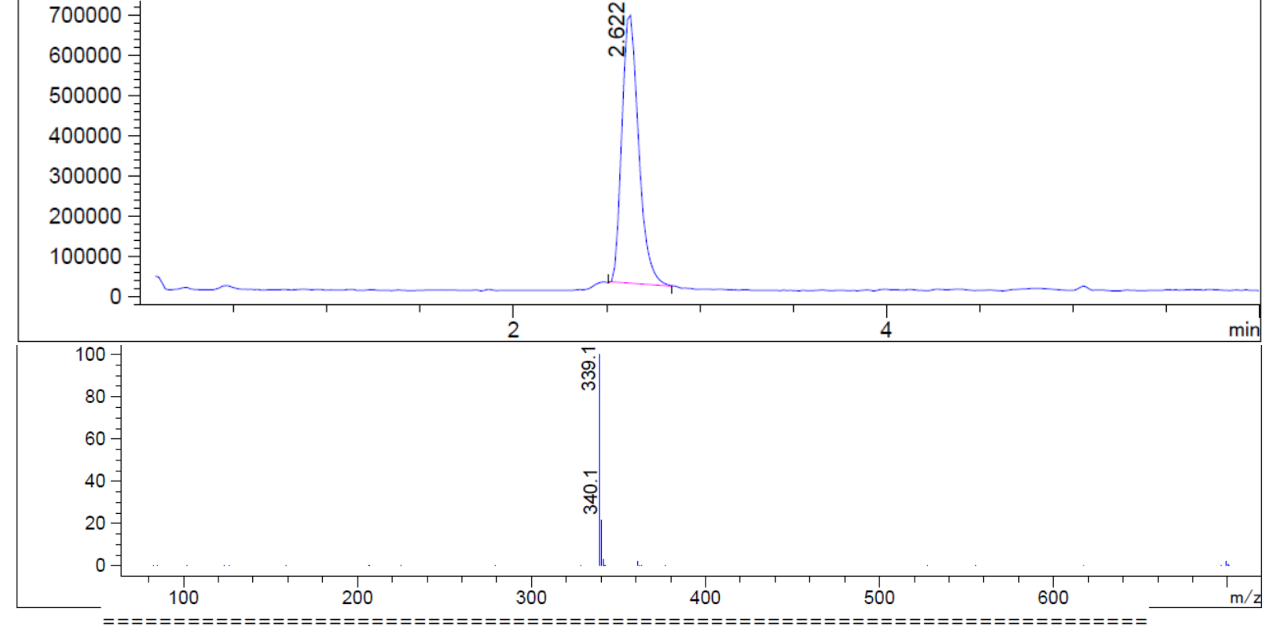


Figure S7. LC/MS spectrometry for compound **3**.


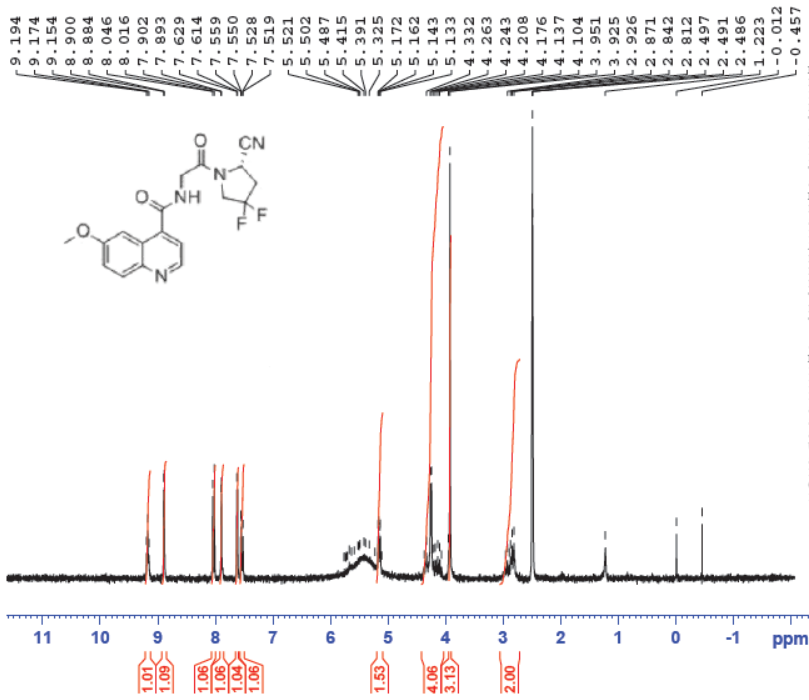


Figure S8. ^1^H NMR spectroscopic data for compound **4**.


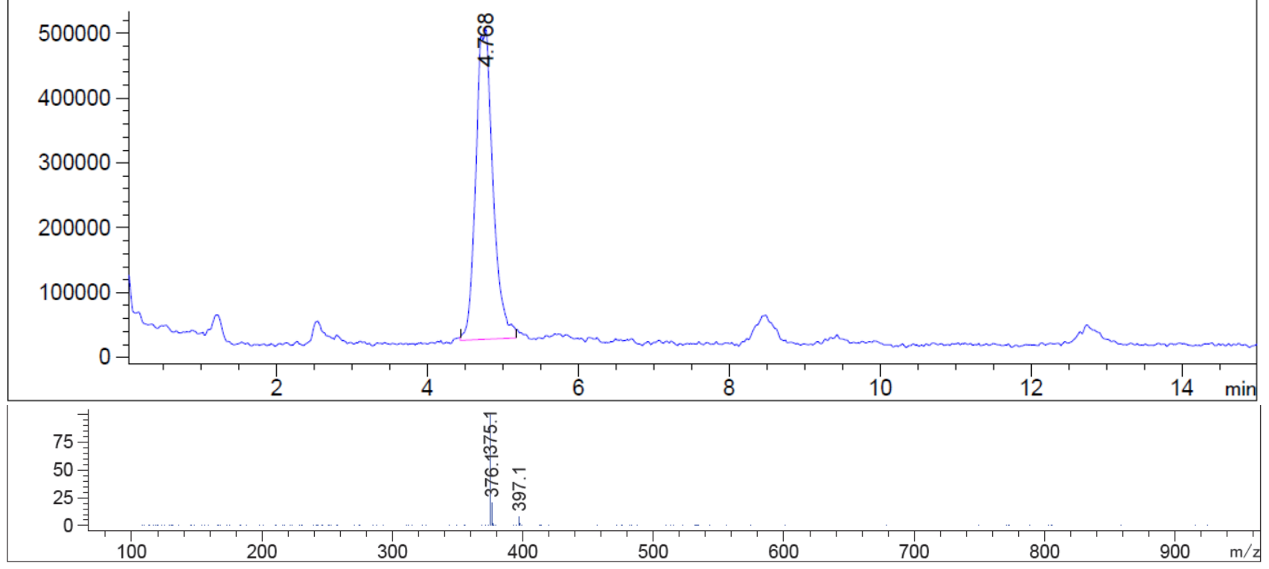


Figure S9. LC/MS spectrometry for compound **4**.


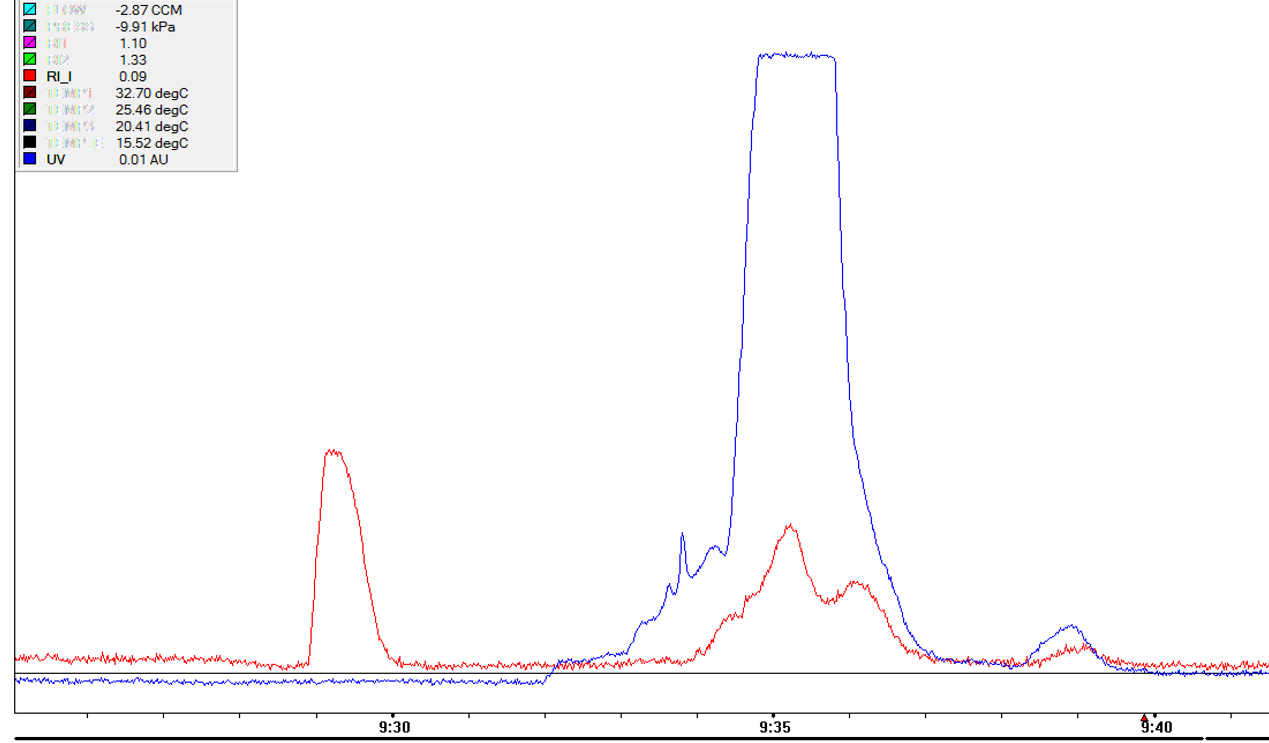


Figure S10. The preparational HPLC data of ^11^C-RJ1101. The HPLC separation conditions: the UV wavelength is 254nm, the Flow rate is 4mLmin^-1^, the mobile phase is *V _acetonitrile_/ V _water_* =32/68. ^11^C-RJ1101 was collected from 9:38 to 9:40, also the retention time was from 10-12min.


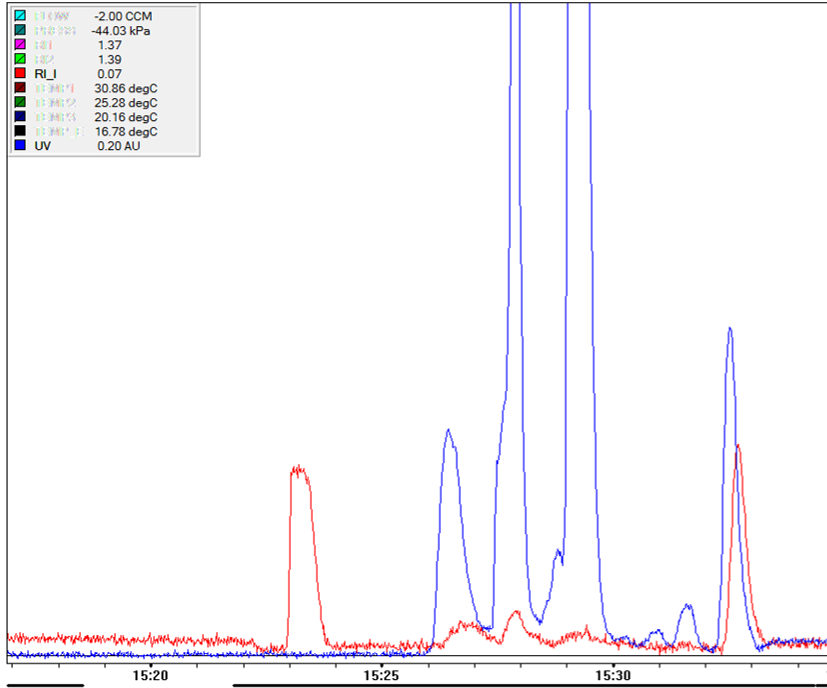


Figure S11. The preparational HPLC data of ^11^C-RJ1102. The HPLC separation conditions: the UV wavelength is 254nm, the Flow rate is 4mLmin^-1^, the mobile phase is *V _acetonitrile_/ V _water_* =42/58. ^11^C-RJ1102 was collected from 15:32 to 15:34, also the retention time was from 9-11min.

**NOTE:** In Figure S10 and Figure S11, the red curve is the radio monitoring curve and the blue curve is the UV monitoring curve. The first radio-peek is the injected monitoring curve also the time point of sample injection.


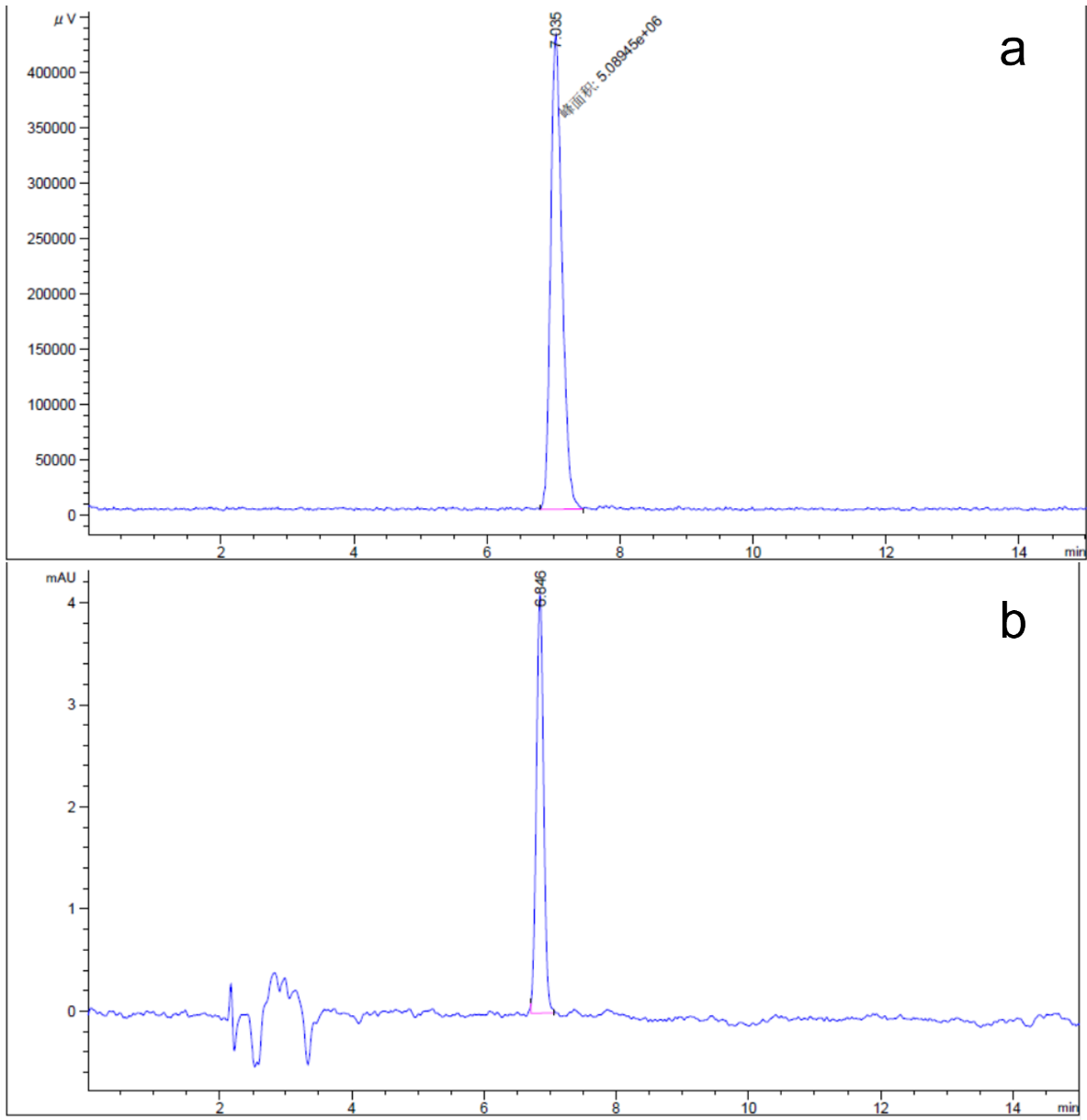


Figure S12. The HPLC analysis of ^11^C-RJ1102. a. the radio-retention time was 7.035min; b. the UV-retention time was 6.846min.

Figure S13. The stability of ^11^C-RJ1102 in PBS at 30, 60, 90, 120, and 150min.


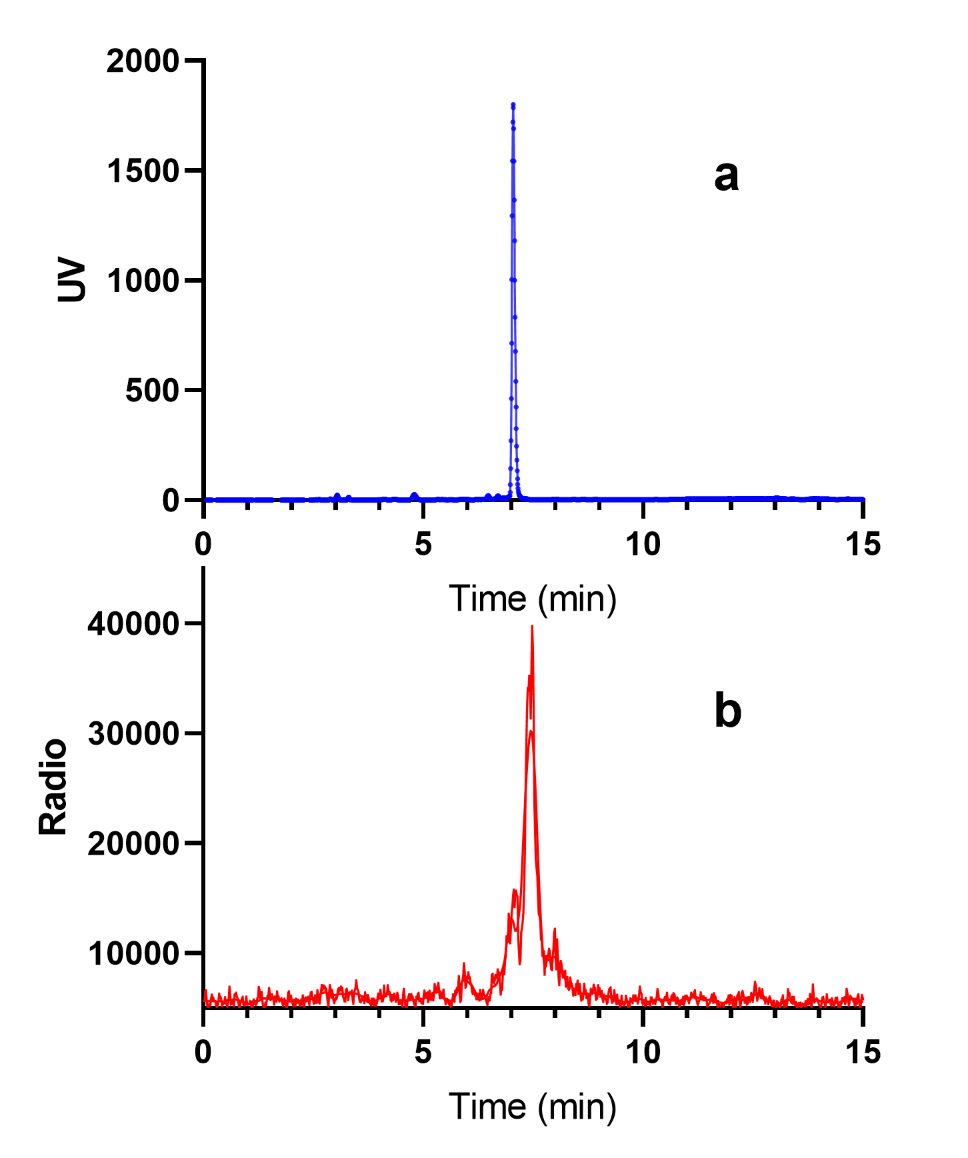


Figure S14. The HPLC of [^68^Ga]Ga-DOTA-FAPI-04 and a represents UV data and b represents Radio data. The retention times were 7.04min and 7.48min, respectively. The chromatographic conditions were described below: flow rate of 1mL/min, UV of 254 nm, injection volume of 20 μL, mobile phase A ( 0.5% trifluoroacetic acid (TFA) aqueous solution), and mobile phase B (acetonitrile), 0-75%B from 0 to 15min.


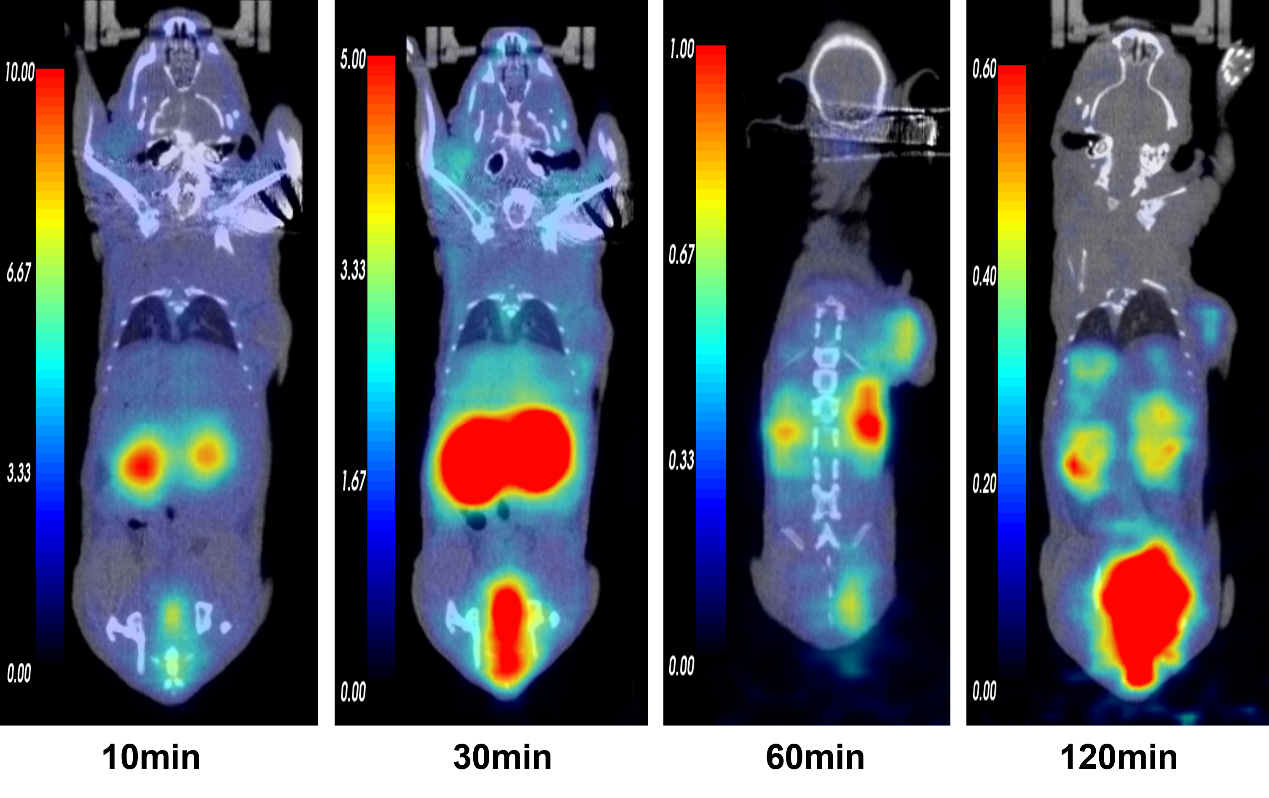


Figure S15. Representative static PET imaging of [^68^Ga]Ga-DOTA-FAPI-04 at 10, 30,60, and 90min.


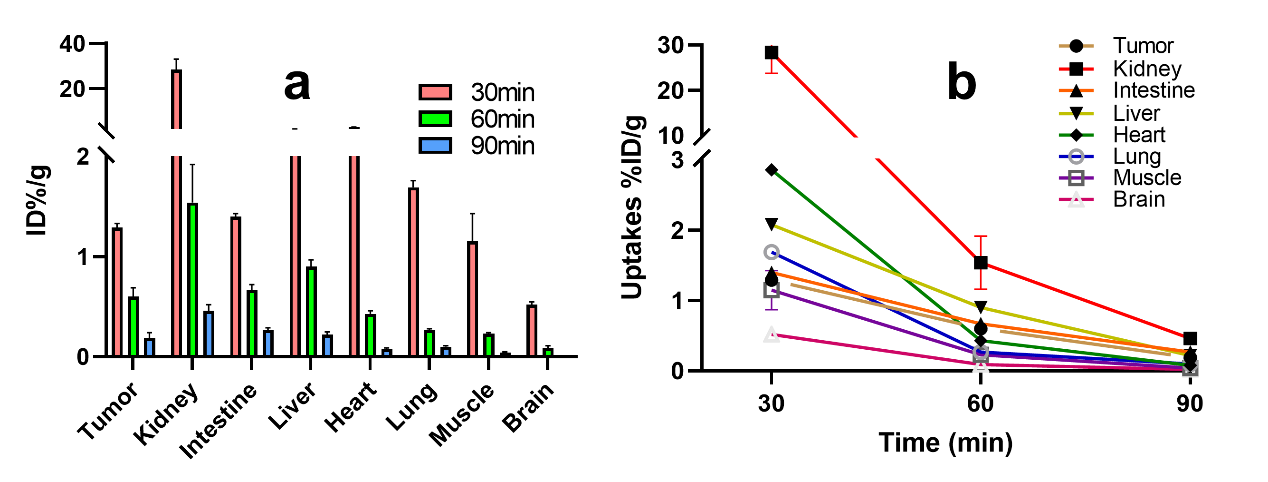


Figure S16. The Representative static PET imaging and distribution of [^68^Ga]Ga-DOTA-FAPI-04. a: The organs or tissues uptakes of [^68^Ga]Ga-DOTA-FAPI-04 in U87MG tumour-bearing nude mice at 30, 60, 90min; b: the time-active-curve (TAC) of [^68^Ga]Ga-DOTA-FAPI-04 at 30, 60, 90min.
